# Use of Preoperative Radiation Therapy in Early-stage and Locally Advanced Breast Cancer

**DOI:** 10.7759/cureus.5748

**Published:** 2019-09-24

**Authors:** Julie L Koenig, Margaret M Kozak, Aaron Sabolch, Kathleen Horst, Jillian Tsai, Irene L Wapnir, Erqi Pollom

**Affiliations:** 1 School of Medicine, Stanford University, Stanford, USA; 2 Radiation Oncology, Stanford Cancer Institute, Stanford, USA; 3 Radiation Oncology, Kaiser Permanente Interstate Radiation Oncology Center, Portland, USA; 4 Radiation Oncology, Stanford University, Stanford, USA; 5 Radiation Oncology, Memorial Sloan Kettering Cancer Center, New York, USA; 6 Surgery, Stanford Cancer Institute, Stanford, USA

**Keywords:** preoperative radiation therapy, neoadjuvant treatment, breast cancer, national cancer database

## Abstract

Purpose

There is growing interest in delivering radiation preoperatively (preopRT) rather than postoperatively (postopRT) for breast cancer. Using the National Cancer Database, we evaluated the use and outcomes of preopRT in breast cancer.

Methods

We identified adult females diagnosed with non-metastatic breast cancer treated with definitive surgery and radiation between 2004 and 2014. Logistic regression models evaluated factors associated with use of preopRT in early-stage (clinical T1-3/N0-1) and locally advanced (clinical T4/N2-3) disease. Rates of breast-conserving surgery, breast reconstruction, positive surgical margins, and 30-day surgical readmissions were compared between patients receiving preopRT and postopRT.

Results

Of 373,595 patients who met our inclusion criteria, 1,245 (0.3%) patients received preopRT. Patients receiving preopRT were more likely to be of lower socioeconomic status and have tumors with higher T stage. Younger age and N1 (vs N0) disease predicted for use of preopRT in early-stage disease, while older age and N0 disease predicted for use of preopRT in the locally advanced setting. PreopRT patients were less likely to undergo breast-conserving surgery and more likely to have positive surgical margins. Rates of unplanned readmissions within 30 days of surgery were similar among patients treated with preopRT and postopRT.

Conclusions

PreopRT is a new treatment strategy for patients with breast cancer with different clinical and sociodemographic drivers of its use in the early-stage and locally advanced settings. We await the results of clinical trials studying the efficacy of this approach.

## Introduction

Many patients with breast cancer are treated with a combination of surgery, chemotherapy, and radiation therapy (RT). Historically, RT has been delivered following surgery and chemotherapy, but there has been growing interest in delivering RT preoperatively. Preoperative RT (preopRT) can potentially downstage tumors, more accurately target tumor, thereby reducing soft tissue toxicity due to smaller treatment volumes [1], improve rates of pathologic complete response (pCR), and facilitate margin-negative resection [2]. Another emerging concept is that of RT as an effective tumor vaccine when directed to intact tumor, activating a robust antitumor immune response and eradicating subclinical disease [3, 4].

While preopRT has been extensively investigated for inoperable and locally advanced breast cancers, there has been less data on the use and efficacy of preopRT for early-stage (T1-3/N0-1) disease. Several recently reported phase I and II trials evaluating the use of preopRT for early-stage breast cancer have shown that this approach is feasible and well-tolerated [1] with good to excellent cosmetic outcomes [5, 6] and local control rates upwards of 90% [6, 7].

Using a large hospital-based national cancer registry, we sought to assess factors associated with receipt of preopRT for early-stage and locally advanced breast cancer, and evaluate the surgical management and outcomes of patients receiving preopRT.

## Materials and methods

Data source

We performed a retrospective cohort study using the National Cancer Database (NCDB) 2014 Participant User File. NCDB is a joint program of the Commission on Cancer (CoC) of the American College of Surgeons and the American Cancer Society (ACS) and is a hospital-based registry with data from more than 1,500 CoC-accredited hospitals. It includes information about demographics, disease stage, and first course of treatment for 70% of newly diagnosed cancer cases in the United States. The CoC and American Cancer Society have not verified and are not responsible for the analytic or statistical methodology used or for the conclusions drawn from these data. This study was exempt from review by our institutional review board.

Patient selection

We identified adult women who were diagnosed with non-metastatic invasive breast cancer between 2004 and 2014. We restricted our study to patients who underwent definitive surgery and preoperative or postoperative external beam RT. Preoperative RT (preopRT) was defined as RT that started within the year prior to definitive surgery based on variables for time of RT and definitive surgery relative to date of diagnosis. Postoperative RT (postopRT) was defined as radiation delivered within six months of surgery. Radiation dose was limited to 14 to 70 Gy based on studies including treatment doses within this range and a small expansion of the range to account for variability in coding [1, 8]. We excluded patients whose RT course extended longer than 10 weeks. Additional inclusion and exclusion criteria are listed in Table 1.

Covariates

Early-stage disease was defined as clinically T1-3/N0-1 and locally advanced was defined as clinically T4/N2-3. Chemotherapy and hormone therapy initiated within one year before definitive surgery were considered neoadjuvant. Adjuvant systemic therapies were similarly defined as therapies initiated within a year after surgery. The NCDB variable for human epidermal growth factor receptor 2 (HER2) was based on information from immunohistochemistry and fluorescence in situ hybridization (FISH) and was consistently available starting in 2010. Results from FISH took precedence when both tests were available. Outcome variables included pathologic complete response (pCR), surgical modality (breast-conserving surgery vs mastectomy), breast reconstruction, surgical margins, and 30-day surgical readmission. NCDB codes pCR based on review of the medical record for a physician statement about response to neoadjuvant therapy. Thirty-day surgical readmission was coded based on an unplanned readmission within 30 days of discharge following hospitalization for resection of the primary cancer.

Statistical analyses

Demographic, tumor, treatment characteristics, and outcomes were compared between patients who received preopRT and postopRT using Chi-square, Fisher’s exact, and Wilcoxon rank sum tests.

Univariate and multivariate logistic regression models were used to determine factors associated with use of preopRT. Our models were adjusted for demographic and tumor factors that were selected a priori. Given that treatment facility type was not available for patients under 40 years old and complete molecular subtype information was not available until after 2010, we constructed separate models additionally adjusting for these factors.

All tests were two-sided with an alpha value of 0.05. Statistical analyses were performed using STATA/SE software (version 14.0, StataCorp, College Station, TX).

## Results

Factors associated with receipt of preoperative RT

We identified 373,595 patients who met our inclusion criteria (Table 1). A total of 1,245 (0.3%) patients received preopRT; 850 (68%) of these patients had early-stage disease (clinical T1-3/N0-1) and 395 (32%) had locally advanced disease (clinical T4/N2-3). Table 2 shows baseline characteristics of patients who received preopRT versus postopRT. In general, patients who received preopRT were more likely to be black, uninsured/Medicaid-insured, living in zip codes of lower median income and educational attainment, and with higher grade and more locally advanced tumors.

**Table 1:** Cohort selection.

	Cohort Selection	No.	%
1	Total breast cancer cases diagnosed from 2004-2014	2,246,280	100.00%
2	Limit to female patients 18 years and older with histologically confirmed non-metastatic, primary invasive breast cancer	1,549,168	68.97%
3	Limit to patients who underwent definitive surgery	1,491,650	66.41%
4	Limit to patients who underwent preoperative or postoperative external beam radiation therapy within one year and six months of surgery, respectively	673,899	30.00%
5	Exclude patients with a prior cancer diagnosis	599,042	26.67%
6	Exclude patients with incomplete clinical staging	439,705	19.57%
7	Exclude patients with incomplete radiation and surgical treatment timing	428,704	19.09%
8	Limit radiation dose to 14 to 70 Gy	388,171	17.28%
9	Exclude patients whose radiation therapy course extended longer than 10 weeks	378,703	16.86%
10	Exclude patients whose diagnosis date precedes reference date to ensure data completeness	373,596	16.63%
11	Exclude patients diagnosed at autopsy	373,595	16.63%

**Table 2:** Baseline patient and tumor characteristics by receipt of preoperative versus postoperative radiation therapy (RT). preopRT: preoperative radiation therapy; postopRT: postoperative radiation therapy; IQR: Interquartile range; HSD: High school degree; AJCC: American Joint Committee on Cancer; T: primary tumor; N: regional lymph nodes; ER: Estrogen receptor; PR: Progesterone receptor; HER2: Human epidermal growth factor receptor 2. ^a^Not available for patients <40 years old. ^b^Includes NCI designated comprehensive cancer centers. ^c^Income and high school degree (HSD) are derived from patient zip code and 2012 American Community Survey data from years 2008-2012. ^d^Molecular subtype was categorized into the following categories: 1) ER/PR+, HER2- (ER or PR positive and HER2 negative), 2) ER/PR+, HER2+ (ER or PR positive and HER2 positive), 3) ER/PR-, HER2+ (ER and PR negative and HER2 positive), and 4) ER/PR-, HER2- (ER, PR, and HER2 negative).

	PostopRT	PreopRT	
Characteristics	No. (%)	No. (%)	p
Total	372,350 (99.7%)	1245 (0.3%)	
Age (years)			<0.001
18-50	91,839 (24.7%)	393 (31.6%)	
51-64	145,778 (39.2%)	500 (40.2%)	
≥65	134,733 (36.2%)	352 (28.3%)	
Age (years), median (IQR)	60 (51, 68)	57 (48, 65)	<0.001
Race			<0.001
White	315,874 (84.8%)	959 (77.0%)	
Black	38,205 (10.3%)	213 (17.1%)	
Other/Unknown	18,271 (4.9%)	73 (5.9%)	
Year of diagnosis			<0.001
2004-2007	63,488 (17.1%)	305 (24.5%)	
2008-2009	71,467 (19.2%)	232 (18.6%)	
2010-2012	134,679 (36.2%)	447 (35.9%)	
2013-2014	102,716 (27.6%)	261 (21.0%)	
Charlson-Deyo Score			0.004
0	320,714 (86.1%)	1108 (89.0%)	
≥1	51,636 (13.9%)	137 (11.0%)	
Insurance Coverage			<0.001
Uninsured	6953 (1.9%)	46 (3.7%)	
Private/Managed Care	214,276 (57.5%)	665 (53.4%)	
Medicare	122,365 (32.9%)	349 (28.0%)	
Medicaid	21,337 (5.7%)	147 (11.8%)	
Unknown/Other	7419 (2.0%)	38 (3.1%)	
Facility type^a^			<0.001
Academic/research^b^	103,244 (27.7%)	395 (31.7%)	
Non-academic	251,782 (67.6%)	745 (59.8%)	
Unknown	17,324 (4.7%)	105 (8.4%)	
Location^a^			<0.001
Northeast	81,240 (21.8%)	223 (17.9%)	
South	110,072 (29.6%)	434 (34.9%)	
Central	100,713 (27.0%)	290 (23.3%)	
West	63,001 (16.9%)	193 (15.5%)	
Unknown	17,324 (4.7%)	105 (8.4%)	
Income^c^			<0.001
< $38,000	49,701 (13.3%)	221 (17.8%)	
≥ $38,000	320,551 (86.1%)	1012 (81.3%)	
Unknown	2098 (0.6%)	12 (1.0%)	
% without HSD^c^			<0.001
≥13%	131,096 (35.2%)	519 (41.7%)	
<13%	239,281 (64.3%)	714 (57.3%)	
Unknown	1973 (0.5%)	12 (1.0%)	
Distance from facility			<0.001
≤50 mi	351,235 (94.3%)	1132 (90.9%)	
>50 mi	19,189 (5.2%)	101 (8.1%)	
Unknown	1926 (0.5%)	12 (1.0%)	
Residence Type			0.085
Metro	313,184 (84.1%)	1036 (83.2%)	
Urban/rural	48,943 (13.1%)	162 (13.0%)	
Unknown	10,223 (2.7%)	47 (3.8%)	
Tumor Size (cm), median (IQR)	1.5 (1.0, 2.5)	2.5 (1.3, 5.0)	<0.001
AJCC Clinical T Stage			<0.001
1	252,738 (67.9%)	487 (39.1%)	
2	87,504 (23.5%)	301 (24.2%)	
3	21,228 (5.7%)	152 (12.2%)	
4	10,880 (2.9%)	305 (24.5%)	
AJCC Clinical N Stage			<0.001
0	306,097 (82.2%)	743 (59.7%)	
1	51,235 (13.8%)	295 (23.7%)	
2	10,097 (2.7%)	132 (10.6%)	
3	4921 (1.3%)	75 (6.0%)	
AJCC Clinical Stage Group			<0.001
Early-stage (T1-3/N0-1)	349,545 (93.9%)	850 (68.3%)	
Locally advanced (T4/N2-3)	22,805 (6.1%)	395 (31.7%)	
Histology			<0.001
Ductal	319,924 (85.9%)	1069 (85.9%)	
Lobular or lobular component	33,396 (9.0%)	84 (6.7%)	
Other	19,030 (5.1%)	92 (7.4%)	
Molecular Subtype^d^			<0.001
ER/PR-,HER2-	25,778 (6.9%)	156 (12.5%)	
ER/PR-,HER2+	7972 (2.1%)	38 (3.1%)	
ER/PR+,HER2-	178,836 (48.0%)	453 (36.4%)	
ER/PR+,HER2+	19,447 (5.2%)	75 (6.0%)	
Unknown/missing	140,317 (37.7%)	523 (42.0%)	
Grade			<0.001
Well differentiated	91,151 (24.5%)	162 (13.0%)	
Moderately differentiated	156,243 (42.0%)	422 (33.9%)	
Poorly, un-differentiated, or anaplastic	103,465 (27.8%)	554 (44.5%)	
Unknown	21,491 (5.8%)	107 (8.6%)	

For both early-stage and locally advanced disease, higher T stage was associated with increased odds of preopRT use. Among early-stage patients, additional factors independently associated with preopRT use were: younger age, lower comorbidity score, higher nodal stage, and high grade (Table 3). In contrast, among patients with locally advanced disease, factors associated with preopRT were older age, less advanced nodal stage, and black race.

**Table 3:** Multivariate logistic regression for the odds of receiving preoperative versus postoperative radiation therapy (RT). preopRT: preoperative radiation therapy; postopRT: postoperative radiation therapy; OR: Odds ratio; CI: Confidence interval; HSD: High school degree; AJCC: American Joint Committee on Cancer; T: primary tumor; N: regional lymph nodes. *Not applicable ^a^Income and high school degree (HSD) are derived from patient zip code and 2012 American Community Survey data from years 2008-2012.

	Early-Stage (T1-3/N0-1)		Locally Advanced (T4/N2-3)	
Characteristic	OR (95% CI)	p	OR (95% CI)	p
Age, years				
18-50	Reference		Reference	
51-64	0.93 (0.79-1.10)	0.381	1.22 (0.95-1.58)	0.122
≥65	0.48 (0.36-0.63)	<0.001	2.15 (1.47-3.13)	<0.001
Race				
White	Reference		Reference	
Black	1.12 (0.91-1.39)	0.283	1.47 (1.13-1.92)	0.004
Other/Unknown	1.13 (0.84-1.52)	0.406	1.35 (0.88-2.10)	0.173
Year of diagnosis				
2004-2007	Reference		Reference	
2008-2009	0.89 (0.71-1.11)	0.304	0.59 (0.43-0.79)	<0.001
2010-2012	0.91 (0.75-1.10)	0.311	0.59 (0.46-0.75)	<0.001
2013-2014	0.76 (0.61-0.93)	0.009	0.40 (0.30-0.55)	<0.001
Charlson-Deyo Score				
0	Reference		Reference	
≥1	0.78 (0.63-0.98)	0.035	0.74 (0.54-1.02)	0.070
Residence type				
Metro	Reference		Reference	
Urban/rural	0.95 (0.77-1.17)	0.599	0.99 (0.74-1.33)	0.958
% without HSD^a^				
≥13%	Reference		Reference	
<13%	0.9 (0.77-1.06)	0.195	1.03 (0.81-1.31)	0.803
Income^a^				
< $38,000	Reference		Reference	
≥ $38,000	0.93 (0.75-1.16)	0.525	0.87 (0.65-1.16)	0.351
Insurance Coverage				
Uninsured	Reference		Reference	
Private/Managed Care	1.07 (0.66-1.74)	0.790	0.55 (0.36-0.84)	0.006
Medicare	1.69 (0.99-2.89)	0.053	0.51 (0.30-0.85)	0.010
Medicaid	1.57 (0.93-2.66)	0.091	0.88 (0.55-1.40)	0.586
Unknown/Other	1.81 (0.98-3.36)	0.058	0.82 (0.39-1.72)	0.594
AJCC Clinical T Stage				
1	Reference		Reference	
2	1.31 (1.10-1.55)	0.002	1.55 (0.78-3.09)	0.216
3	2.33 (1.83-2.96)	<0.001	2.14 (1.05-4.34)	0.035
4	*	*	6.91 (3.60-13.26)	<0.001
AJCC Clinical N Stage				
0	Reference		Reference	
1	1.26 (1.05-1.52)	0.014	0.57 (0.42-0.77)	<0.001
2	*	*	1.02 (0.75-1.39)	0.889
3	*	*	1.13 (0.80-1.59)	0.483
Histology				
Ductal	Reference		Reference	
Lobular	0.99 (0.76-1.28)	0.930	0.47 (0.27-0.82)	0.008
Other	1.28 (0.96-1.71)	0.098	2.05 (1.45-2.90)	<0.001
Grade				
Well differentiated	Reference		Reference	
Moderately differentiated	1.18 (0.96-1.45)	0.109	1.00 (0.61-1.64)	0.989
Poorly differentiated	1.69 (1.36-2.09)	<0.001	0.98 (0.60-1.59)	0.925
Unknown	1.70 (1.25-2.30)	0.001	1.07 (0.61-1.88)	0.816

In models adjusting for treatment facility type, treatment at an academic facility was associated with greater likelihood of undergoing preopRT among early-stage patients (Odds ratio [OR], 1.23; 95% confidence interval [CI], 1.05-1.43; p = 0.009), but not for those with locally advanced disease (OR, 1.10; 95% CI, 0.87-1.38; p = 0.437). On multivariate analysis of patients with known molecular subtype, patients with estrogen receptor (ER)/progesterone receptor (PR)-, HER2- (OR, 1.30; 95% CI, 1.05-1.60; p = 0.018) tumors were more likely to receive preopRT relative to patients with ER/PR+, HER2- tumors.

Treatment characteristics

For early-stage disease, patients treated with preopRT versus postopRT were more likely to receive neoadjuvant chemotherapy (NACT; 47.2% vs 13.4%) and neoadjuvant hormonal therapy (NAHT; 37.4% vs 1.7%; Table 4). Patients treated with preopRT versus postopRT for locally advanced disease were equally as likely to receive NACT (74.4% vs 73.2%) but were more likely to receive NAHT (30.6% vs 6.9%). Among 695 patients who received preopRT and NACT, 95.0% received preopRT after NACT (early-stage: 96.0% vs locally advanced: 93.5%; p = 0.141). Overall, 33,716 (9.0%) patients received no systemic therapy including 81 patients who received preopRT. Among patients who received preopRT, patients treated without systemic therapy tended to be older (median age 69 vs 56 years; p < 0.001) and have early-stage disease (80.2% vs 67.4%; p = 0.017).

**Table 4:** Systemic therapies received within one year of definitive surgery by patients treated with preoperative versus postoperative radiation therapy (RT) in early-stage (T1-3/N0-1) and locally advanced (T4/N2-3) breast cancer. preopRT: preoperative radiation therapy; postopRT: postoperative radiation therapy. *In order to protect patient identity, cells with fewer than 11 patients are not shown.

	Early-Stage (T1-3/N0-1)			Locally Advanced (T4/N2-3)		
	PostopRT	PreopRT		PostopRT	PreopRT	
Characteristics	No. (%)	No. (%)	p	No. (%)	No. (%)	p
Total	349,545	850		22,805	395	
Chemotherapy			<0.001			<0.001
None	213,100 (61.0%)	308 (36.2%)		1838 (8.1%)	58 (14.7%)	
Neoadjuvant	46,818 (13.4%)	401 (47.2%)		16,704 (73.2%)	294 (74.4%)	
Adjuvant	82,520 (23.6%)	42 (4.9%)		3540 (15.5%)	<11	
Unknown	7107 (2.0%)	99 (11.6%)		723 (3.2%)	≥11	
Hormone therapy			<0.001			<0.001
None	77,742 (22.2%)	274 (32.2%)		8545 (37.5%)	188 (47.6%)	
Neoadjuvant	6079 (1.7%)	318 (37.4%)		1568 (6.9%)	121 (30.6%)	
Adjuvant	242,517 (69.4%)	189 (22.2%)		11,373 (49.9%)	56 (14.2%)	
Unknown	23,207 (6.6%)	69 (8.1%)		1319 (5.8%)	30 (7.6%)	

Patients treated with preopRT received a median radiation dose of 59 Gy (interquartile range [IQR], 50-60.4 Gy) over a median of 43 days (IQR, 35-49 days). Among those who did not additionally receive postopRT (1,088 patients; 87.4%), preopRT was completed a median of 70 days (IQR, 37-143 days) before surgery. Of patients with known radiation fields, 73.9% of early-stage patients had preopRT to the breast/chest wall only and 26.1% had preopRT to the breast/chest wall and lymph nodes. Among locally advanced patients, 40.4% had preopRT to the breast/chest wall only and 59.6% had preopRT to the breast/chest wall and lymph nodes.

Surgical management and outcomes

Patients who received preopRT were less likely to undergo breast-conserving surgery (Table 5). Compared to patients who received postopRT, patients who received preopRT were more likely to have post-mastectomy reconstruction in early-stage disease, but less likely to have reconstruction in locally advanced disease. Patients who received preopRT had a higher rate of positive surgical margins in both early-stage (6.0% vs 4.0%; p = 0.004) and locally advanced disease (13.1% vs 7.7%; p < 0.001). Rates of unplanned readmissions within 30 days of surgery were similar among patients treated with preopRT versus postopRT (1.2% vs 1.2%; p = 0.894).

**Table 5:** Surgical management and outcomes of patients receiving preoperative versus postoperative radiation therapy (RT) in early-stage (T1-3/N0-1) and locally advanced (T4/N2-3) breast cancer. preopRT: preoperative radiation therapy; postopRT: postoperative radiation therapy. *In order to protect patient identity, cells with fewer than 11 patients are not shown. #Fisher’s exact test ^a^Unplanned readmission to the treating hospital within 30 days of discharge following hospitalization for resection of the primary cancer.

	Total			Early-Stage (T1-3/N0-1)			Locally Advanced (T4/N2-3)		
	PostopRT	PreopRT		PostopRT	PreopRT		PostopRT	PreopRT	
	n (%)	n (%)	p	n (%)	n (%)	p	n (%)	n (%)	p
Breast conserving surgery (vs mastectomy)	307,322 of 372,350 (82.5%)	544 of 1,245 (43.7%)	<0.001	302,410 of 349,545 (86.5%)	491 of 850 (57.8%)	<0.001	4,912 of 22,805 (21.5%)	53 of 395 (13.4%)	<0.001
Breast reconstruction	18,939 of 372,350 (33.2%)	168 of 1,245 (28.7%)	0.020	14,344 of 349,545 (34.5%)	131 of 850 (44.1%)	<0.001	4,595 of 22,805 (29.7%)	37 of 395 (12.8%)	<0.001
Positive (vs negative) surgical margins	15,613 of 369,928 (4.2%)	99 of 1,213 (8.2%)	<0.001	13,891 of 347,473 (4.0%)	50 of 839 (6.0%)	0.004	1,722 of 22,455 (7.7%)	49 of 374 (13.1%)	<0.001
Readmission within 30 days of surgical discharge^a^	4,392 of 361,952 (1.2%)	15 of 1,201 (1.2%)	0.894^#^	4,085 of 339,966 (1.2%)	<11	1.000^#^	307 of 21,986 (1.4%)	<11	0.661^#^

In total, 14.8% of preopRT patients had response to neoadjuvant therapy recorded in the database with an overall pathologic complete response (pCR) rate of 25.5%. Of the 90 early-stage and 94 locally advanced patients who had preopRT with recorded pCR, pCR rates were 32.2% and 19.1%, respectively. Among 83 patients who had preopRT, ER/PR+, HER2- tumors, and pCR recorded in the database, pCR rate was 18.1%. Of the 46 early-stage and 37 locally advanced patients who had preopRT and ER/PR+, HER2- tumors with recorded pCR, pCR rates were 23.9% and 10.8%, respectively.

## Discussion

The traditional paradigm of surgery followed by possible RT and/or chemotherapy for patients with breast cancer has been challenged by the increased use of neoadjuvant systemic therapy and interest in preopRT [8]. Our analysis evaluating patterns of care with preopRT in the U.S. through a large hospital-based registry of breast cancer patients contributes to the growing literature on preopRT in breast cancer, which up until now has been primarily comprised of single institution experiences. We characterize several distinct patient populations that have received preopRT thereby furthering our understanding of its potential applications.

We found that use of preopRT varied by demographic and tumor characteristics, suggesting there may be different indications for its use across treatment settings. Patients undergoing preopRT were more likely to be younger, black, and uninsured or Medicaid-insured. Furthermore, these patients were more likely to present with T3-4 tumors, more advanced nodal disease, and triple negative subtype. These findings build upon previously published literature highlighting socioeconomic disparities among breast cancer patients [9, 10]; patients of lower socioeconomic status were likely selected for neoadjuvant treatment with preopRT due to presentation with more advanced disease. Younger age and nodal involvement (N1 vs N0) predicted for use of preopRT in the setting of early-stage disease, but older age and N0 disease predicted for use of preopRT in patients with locally advanced disease. It may be that younger women with early-stage disease and positive nodes undergo preopRT to de-escalate surgery of the breast and axilla similar to the emerging role of NACT in this setting [11]. PreopRT may be an alternative to NACT or an adjunct to NAHT in older, locally advanced patients with negative nodes, since endocrine therapy is associated with slow, partial tumor responses [12]. PreopRT may also be used in patients demonstrating less of a response to NACT, as we found 95% of patients who received NACT and preopRT underwent preopRT after NACT [8]. Finally, we found that treatment at an academic facility was associated with use of preopRT in early-stage disease, but not locally advanced disease, which highlights the novelty of this approach in early-stage breast cancer.

Given that women who undergo breast conservation have reported higher satisfaction with body image than women who undergo mastectomy, a neoadjuvant strategy that facilitates breast-conserving treatment is important [13]. However, our analysis does not inform us regarding the reasons preopRT was chosen among patients. The significantly lower rates of breast conservation, 57.8% vs 86.5%, for the early-stage preopRT group may be due to presentation with relatively more advanced disease, sociodemographic drivers of treatment decisions, patient preferences, and surgeon recommendations [14]. Other factors that are relevant when discussing breast conservation include whether neoadjuvant treatment can improve rates of margin-negative resection and pCR. In the NACT setting, no standard approach to breast conservation and margin interpretation has been delineated. There are conflicting reports about whether NACT is associated with lower [2, 15] or higher [16] rates of re-excision following lumpectomy. We found that preopRT was associated with higher rates of positive surgical margins (8.2% vs 4.2%). These results may be attributed to selection bias, as preopRT may have been motivated by poor response to NACT or NAHT, lesion location in the breast, or size of the lesion with respect to the breast. However, a heterogeneous tumor response with mosaic patterns of tumor shrinkage could have contributed to greater uncertainty during surgical resection. Additionally, depending on the timing of preopRT with respect to operative intervention, the degree of tissue inflammation or induration encountered could have further impaired surgical excision. Further work is needed to establish the optimal timing of surgery after preopRT [1, 6, 7, 17].

In our analysis, 25% of patients receiving preopRT achieved pCR (32% early-stage and 19% locally advanced). Among patients with ER/PR+, HER2- tumors, 18% of patients receiving preopRT achieved pCR (24% early-stage and 11% locally advanced). In contrast, among patients with low-grade, hormone receptor-positive tumors, the rate of pCR following NACT and/or NAHT alone is low, ranging from 2 to 10% [18, 19]. Furthermore, pCR is not a strong prognostic marker among these patients [20]. Although our pCR analysis was limited by the low number of patients with this data available, these favorable pCR rates raise the intriguing possibility of the potential role of preopRT to increase pCR and as a tool of bioselection analogous to the emerging role that NACT plays in patients with hormone receptor-negative disease.

Other studies of preopRT have reported lower rates of pCR. Nichols et al. conducted a prospective trial of preoperative accelerated partial breast irradiation in patients with early-stage breast cancer and found a pCR rate of 15% among patients with ER+ tumors [6]. However, no patients on this trial received NACT or NAHT. In the locally advanced setting, Adams et al. showed that pCR was associated with significantly better disease-free and overall survival following preoperative concurrent paclitaxel-radiation [21].

In addition to improving rates of breast-conserving surgery, there is interest in using preopRT to facilitate immediate breast reconstruction. PreopRT followed by combined oncologic and reconstructive surgery may be preferable to traditional sequencing with postopRT because it may reduce the shrinkage of transferred tissue and affect perfusion or contracture around implants [8]. We found higher rates of reconstruction among early-stage preopRT patients and lower rates of reconstruction among locally advanced preopRT patients; however, these rates may be driven by sociodemographic rather than tumor characteristics [22-24].

Lastly, we found that preopRT was not associated with increased risk of readmission within 30 days of surgical discharge. This finding is corroborated by multiple studies demonstrating acceptable postoperative complication rates among breast cancer patients receiving preopRT prior to mastectomy with or without immediate breast reconstruction [25-27].

To our knowledge, this is the first nationwide study to detail the use of preopRT in newly diagnosed breast cancer patients. The strengths of this analysis are that it encompasses a contemporary population from a hospital-based registry reflective of the U.S. population. Our conclusions are confounded by indication, missing/unknown data such as molecular subtype, lack of pCR coding in the NCDB for greater than 90% of patients, and no specification regarding the use of partial breast irradiation. We also could not account for unmeasured confounders such as genetic testing, family history, patient preferences, and provider recommendations. Our assessment of complications is limited to 30-day surgical readmissions and does not account for late complications or less severe complications not requiring hospital admission. Lastly, our results may only apply to women treated at hospitals accredited by the Commission on Cancer and the American Cancer Society, which are enriched for hospitals with strong oncology services [28].

## Conclusions

The role of preopRT is being reconsidered for patients with breast cancer with different clinical and sociodemographic drivers of its use in the early-stage and locally advanced settings. This approach may improve rates of pCR in hormone receptor-positive tumors and facilitate breast reconstruction in early-stage disease. We await the results of ongoing trials that will shed further light on the feasibility and effectiveness of preopRT.
